# Supplementary material for: DMRforPairs: identifying Differentially Methylated Regions between unique samples using array based methylation profiles
Source: BMC Bioinformatics. 2014 May 15;15:141. doi: 10.1186/1471-2105-15-141 (PMC4046028; doi:10.1186/1471-2105-15-141)
Supplement: Additional file 2 — DMRforPairs output for the comparison of A431-MCF7 and NA17018-NA17105. Please start from the HTML files in each folder. Available via the BMC Bioinformatics website. [file 1471-2105-15-141-S2.zip › 1394847754114233_MOESM2_ESM/A431_MCF7/figures/7390.pdf]

RegionID: 7390, chr11:64014518−64014598−M_values

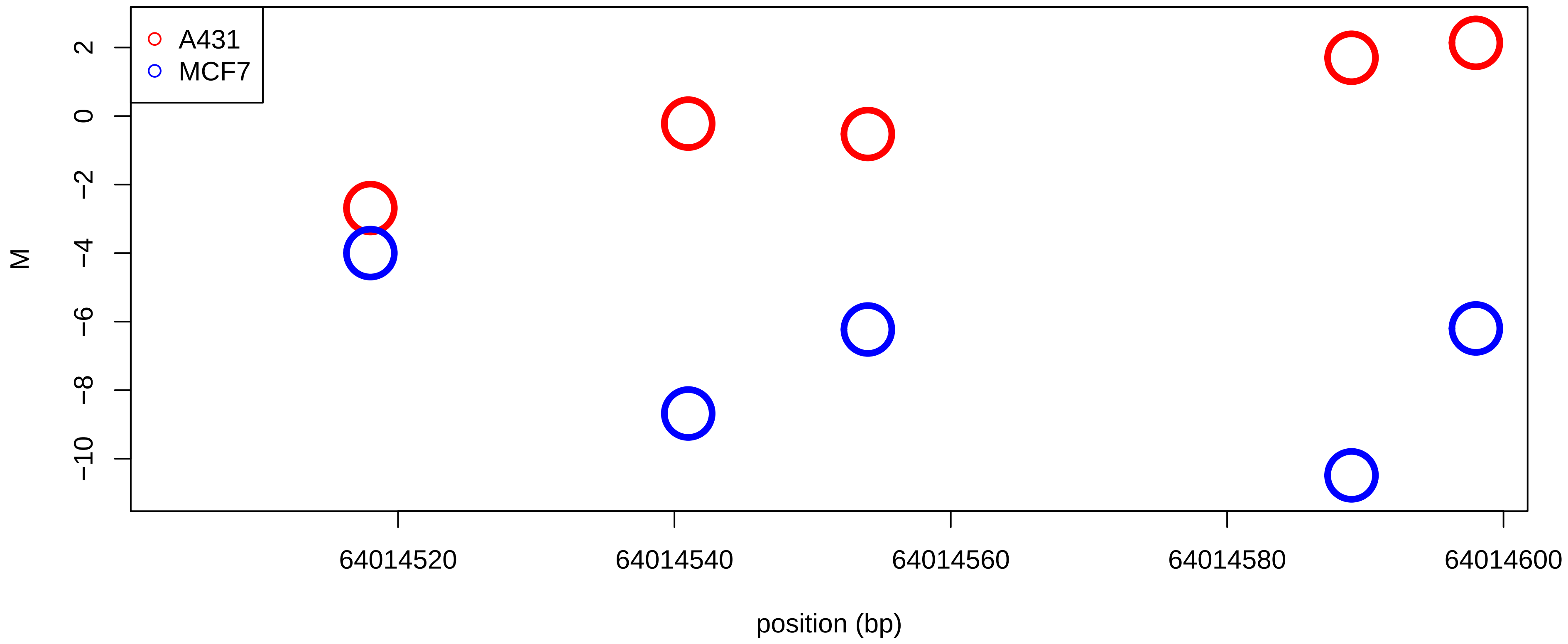

RegionID: 7390, chr11:64014518−64014598−Beta_values

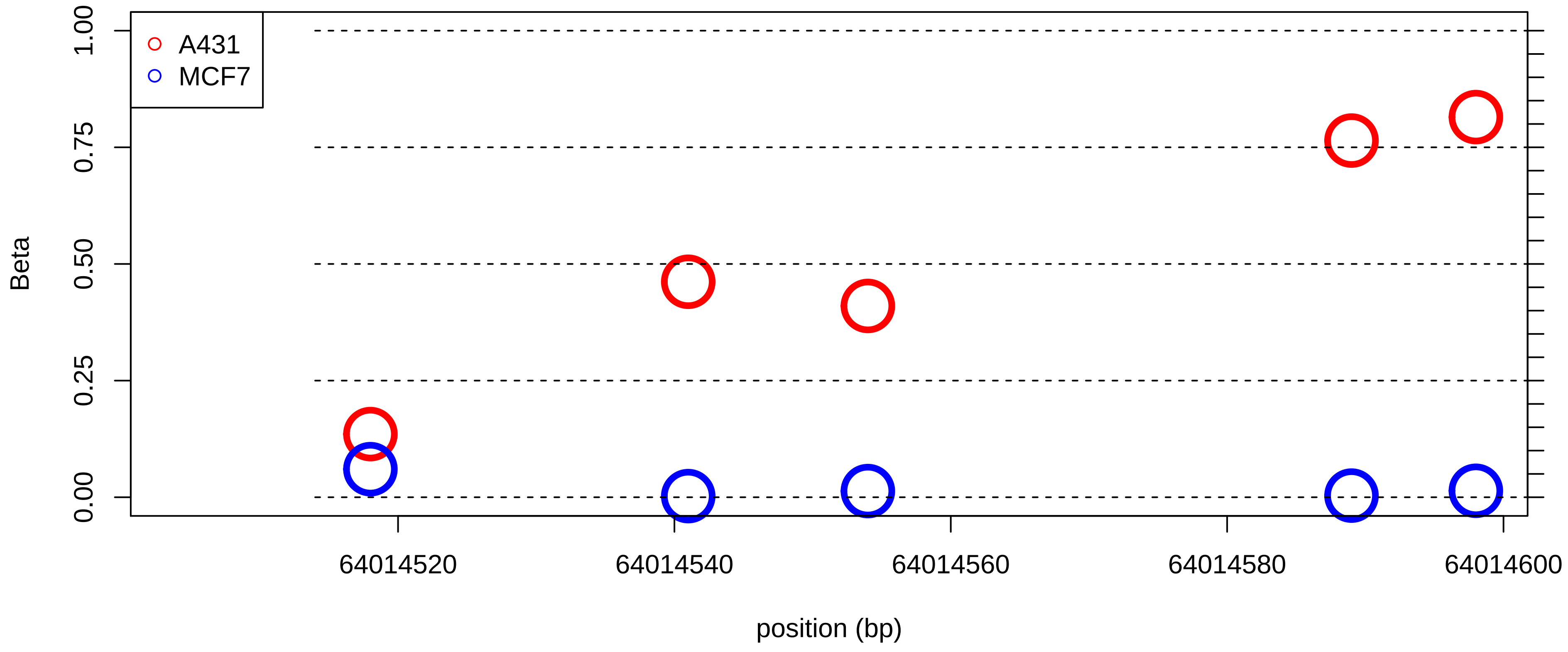

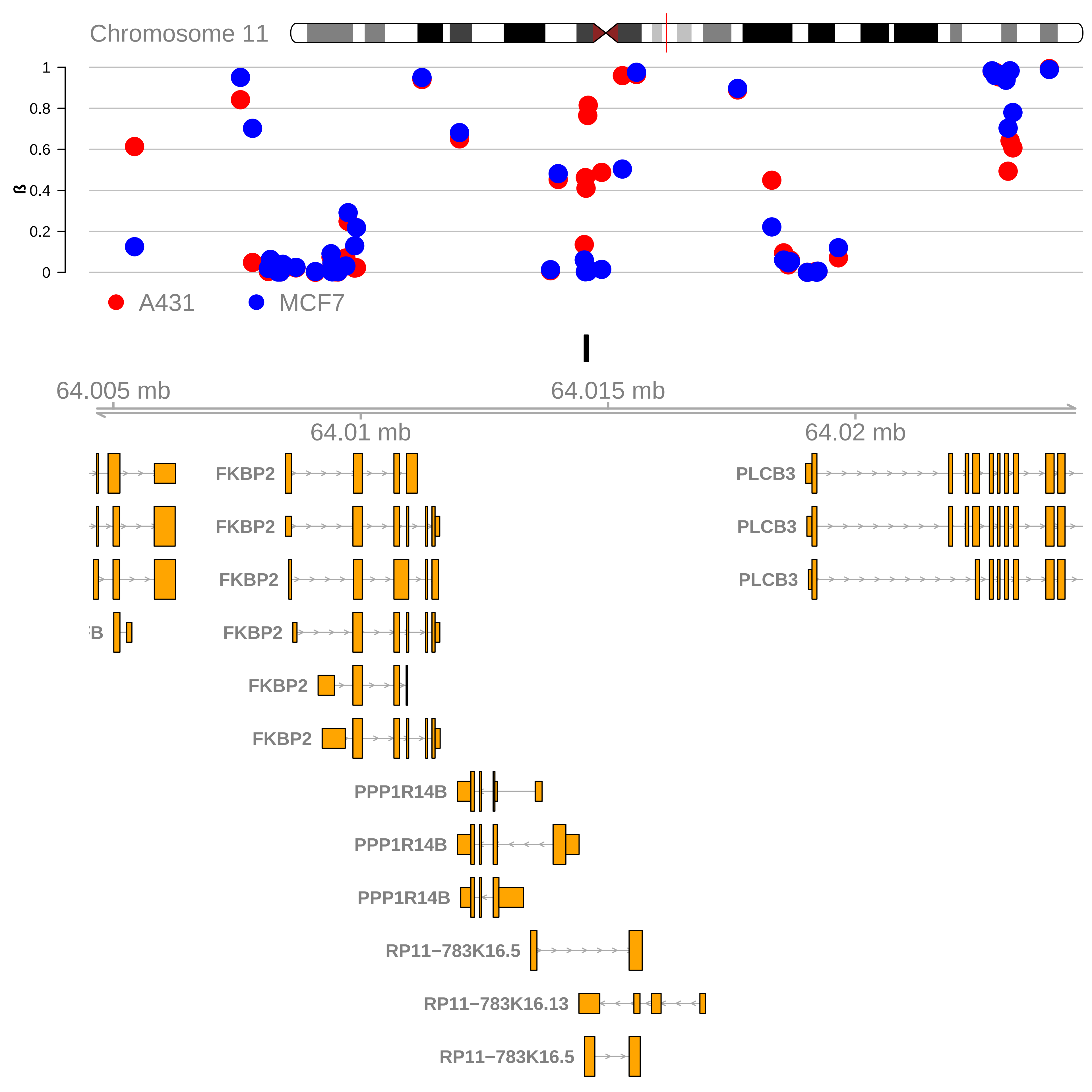
Chromosome 11
ß
1
0.8
0.6
0.4
0.2
0
A431
MCF7
64.005 mb
64.015 mb
64.01 mb
64.02 mb
FKBP2
FKBP2
FKBP2
FKBP2
FKBP2
FKBP2
PLCB3
PLCB3
PLCB3
PPP1R14B
PPP1R14B
PPP1R14B
RP11−783K16.5
RP11−783K16.13
RP11−783K16.5
